# Process and Formulation Parameters Governing Polymeric Microparticle Formation via Sequential NanoPrecipitation (SNaP)

**DOI:** 10.1021/acsengineeringau.5c00035

**Published:** 2025-07-04

**Authors:** Parker K. Lewis, Nouha El Amri, Erica E. Burnham, Natalia Arrus, Nathalie M. Pinkerton

**Affiliations:** 1 Department of Chemical and Biomolecular Engineering, Tandon School of Engineering, New York University, Brooklyn 11201, United States; 2 Department of Biomedical Engineering, Tandon School of Engineering, New York University, Brooklyn 11201, United States; 3 Department of Mechanical and Aerospace Engineering, Tandon School of Engineering, New York University, Brooklyn 11201, United States

**Keywords:** drug delivery, microparticle, sequential nanoprecipitation, continuous flow manufacturing, polymer microparticle

## Abstract

Polymeric microparticles (MPs) are valuable drug delivery vehicles for extended-release applications, but current manufacturing techniques present significant challenges in balancing size control with scalability. Industrial synthesis processes provide high throughput but limited precision, while laboratory-scale technologies offer precise control but poor scalability. This study explores Sequential NanoPrecipitation (SNaP), a two-step controlled precipitation process for polymeric microparticle production, to bridge the gap between laboratory precision and industrial scalability. We systematically investigated critical process parameters governing MP formation, focusing on poly­(lactic acid) (PLA) MPs stabilized with poly­(vinyl alcohol) (PVA). By comparing vortex and impinging jet mixing geometries, we demonstrated that vortex mixing provides superior performance for core assembly, particularly at higher polymer concentrations. We established the influence of delay time (*T*_d_) and core stream concentration (*C*_core_) on particle size, confirming that microparticle assembly follows Smoluchowski diffusion-limited growth kinetics within defined operational boundaries. Through this approach, we achieved precise control over microparticle size (1.6–3.0 μm) with narrow polydispersity. The versatility of SNaP was further demonstrated by the successful formation of MPs with different stabilizers while maintaining consistent size control. Finally, we validated the pharmaceutical relevance of SNaP by encapsulating itraconazole with high efficiency (83–85%) and characterizing its sustained release profile. These findings establish SNaP as a robust, scalable platform for high-quality pharmaceutical microparticle production with superior control over critical quality attributes.

## Graphical abstract

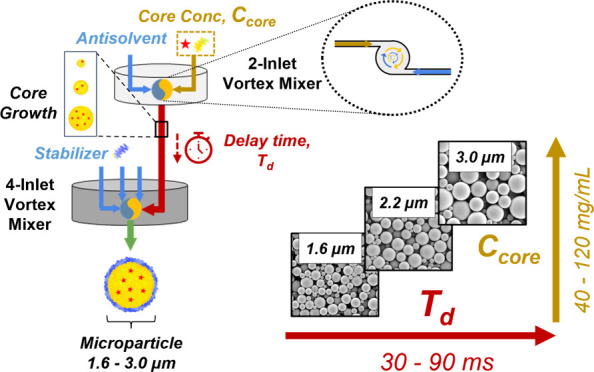

## 1 Introduction

Polymeric microparticles (MPs) have emerged as important drug delivery vehicles, particularly for extended-release parenteral formulations, inhalable formulations and vaccines.^1–5^ Their biocompatibility, favorable tissue retention, and controllable release kinetics make them well-suited for depot applications.^3,4,6,7^ Commercial successes include extended-release parenteral formulations such as naltrexone (Vivitrol) for opioid addiction, risperidone (Risperdal Consta) for schizophrenia, and metoprolol succinate (Betaloc ZOK) for angina.^8–10^ Despite these achievements, pharmaceutical MP production faces significant challenges across scales. Industrial processes like spray drying and emulsification suffer from limited control over particle size and uniformity.^4,11^ Moreover, scaling down spray drying to small batch sizes for formulation development proves technically difficult.^12^ Conversely, newer laboratory technologies such as microfluidics and membrane emulsification excel at precise size control and small-scale operation but face severe scale-up limitations due to their inherently low throughput capacities (<10 mL/min).^12–17^ This dichotomy creates a critical gap between laboratory-scale precision and industrial-scale production capacity.

The nascent Sequential NanoPrecipitation (SNaP) process has the potential to bridge this gap. SNaP is a two-step controlled precipitation process that enables the production of polymeric particles ranging from nanometers to microns in size with narrow polydispersities.^18,19^ SNaP leverages the same rapid micromixing and hydrophobic-driven particle self-assembly principles as the scalable Flash NanoPrecipitation (FNP) process.^20,21^ However, unlike FNP which assembles nanoparticles in one step,^21^ in SNaP, the particle core formation and stabilization are decoupled, enabling enhanced control over particle assembly and unlocking the previously unattainable micron particle size scale.^18^ This is achieved by performing micromixing in series under continuous flow (Figure 1). In the first mixing step, dissolved core materials are rapidly mixed with antisolvent under turbulent conditions (Re > 2000) to achieve micromixing, followed by the induction of nucleation and core growth. In the second micromixing step, stabilizer is introduced to arrest particle growth. By tuning the delay time (*T*_d_) between the first and second mixing steps, we can control the core growth time and hence particle size. Moreover, SNaP can controllably generate composite inorganic–organic nanoparticles and nanoparticles with extremely high drug loadings (>70 wt % drug).^19,22^ A key advantage of SNaP is its flexibility in scale: it can be run with small micromixers and low minimum stream volumes required to simulate continuous flow (<3 mL per stream required when using a 60 mL/min stream flow rate) for formulation development with minimal material requirements as we will show herein, and also has the potential to be scaled-up with larger micromixers for continuous flow production at industrial rates (>5 L/min total output rate).^23,24^

**Figure 1:**
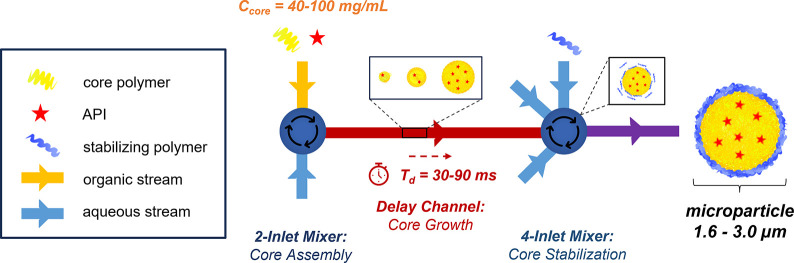
**Schematic of the SNaP process.** An organic stream containing the microparticle core components is rapidly mixed against an antisolvent in the first micromixing step to initiate the core formation. The outlet is connected to a second micromixer, which introduces the stabilizer and arrests the microparticle growth. The delay time between the first and second mixing steps controls the time of the core growth.

In this study, we systematically investigated SNaP process parameters and formulation variables to elucidate the governing principles of MP property control. We employed SNaP to synthesize spherical polymeric MPs comprising industry-standard components: hydrophobic polylactic acid (PLA) cores stabilized via hydrophilic poly­(vinyl alcohol) (PVA). We first investigated the core assembly mixing step by evaluating the importance of mixing geometry for uniform particle assembly. We subsequently explore the process parameters of *T*_d_ and core stream solids concentration (C_core_), establishing the variable parameter space, identifying regions of tunable particle assembly, and determining the boundaries of process stability. Through this systematic approach, we demonstrate precise and reproducible size control of monodisperse particles within the 1.6–3.0 μm diameter range, ideal for inhalation delivery applications. We go on to show consistency in PLA MP size across different stabilizing polymers. Finally, we validate the drug loading capability of the system through reproducible, high-efficiency encapsulation of itraconazole and characterize the MP drug release rate. With these findings, we present the SNaP process as a promising new standard for high quality production of polymeric MPs.

## 2 Materials & Methods

### 2.1 Materials

Tetrahydrofuran (THF, HPLC grade), acetonitrile (ACN, HPLC grade) were purchased from Fisher Scientific (USA). Rubrene (98%), poly­(vinyl alcohol) (PVA, 13–23 kDa), polyvinylpyrrolidone (PVP, 40 kDa) and Tween-80 (ultrapure) were purchased from Sigma-Aldrich (USA). Polyethylene glycol polylactic acid block copolymer (PEG–PLA, 5kD-5kD) was purchased from Evonik Inc. (Germany). Itraconazole (98%) was purchased from TCI America. Ultrapure water (18.2 MΩ·cm) was generated by a MilliporeSigma Milli-Q Water Purification System (USA). Polylactic acid homopolymer (PLA, 10–18 kDa) was synthesized in-house according to the protocol described in Section 2.2.1. For mixer assembly, Tefzel tubing (0.040” & 0.060” ID), female LuerTight syringe fitting systems (1/16” OD), VacuTight Fittings (1/4–28 – 1/8), and flangeless male nut fittings (1/4–28, 1/16”) were purchased from Idex Health and Science (USA). O-rings (75 Viton, 1.5 × 35 mm) and heated inserts for 3D printed mixer inlets were purchased from McMaster Carr (USA).

For polymer synthesis, 2-hydroxyethyl bromoisobutyrate (95%), 1,8-diazabicyclo(5.4.0)­undec-7-ene (95%), methanol (ACS grade), and deuterated chloroform were purchased from Sigma-Aldrich (USA). Chloroform (anhydrous, extra dry) and octadecyltrichlorosilane were purchased from Thermo Scientific (USA). Toluene (ACS grade) and chloroform (HPLC grade) were purchased from Alfa Aesar (USA). Dl-lactide (99%) was purchased from Beantown Chemical (USA). Hydrochloric acid (37%) was purchased from Acros Organics (USA).

### 2.2 Methods

#### 2.2.1 Synthesis and Characterization of PLA

Dl-lactide was added (12.6 g, 87.6 mmol) to a flame-dried, silanzied 200 mL round-bottom flask equipped with a large stir bar before undergoing three vacuum/nitrogen purge cycles followed by overnight drying under high vacuum. In a glovebox, DBU (1.12 mmol) was added to a flame-dried pear-shaped flask equipped with a stir bar. Anhydrous chloroform, extra dry over molecular sieves, was then used to dilute the monomer and catalyst with 82 and 18 mL, respectively. The monomer solution was then heated to 50 °C using a mineral oil bath. The initiator, HeBriB (1.19 mmol), was then added to the monomer solution and allowed to stir for 15 min. To initiate the reaction, the catalyst solution was transferred via cannula to the monomer solution for a final reaction molarity of 0.9 M. Following initiation, the heat was immediately turned off, and the reaction solution was allowed to remain in the cooling mineral oil under nitrogen. The reaction was terminated after 2 h by extracting the catalyst with 1 M HCl_(aq)_ followed by two brine washes. The organic phase was then concentrated and precipitated over 30x excess ice-cold methanol to isolate the PLA. The product was transferred to silanzied scintillation vials and dried overnight under high vacuum to yield a crystalline, white powder (12.2 g, 94.4% yield, Đ = 1.18).

Polymer characterization was performed by ^1^H NMR and gel permeation chromatography (GPC). ^1^H NMR spectra were acquired on a Bruker AVANCE NEO 500 MHz instrument with minimum 32 scans and a 10-s relaxation delay time. ^1^H NMR (500 MHz, CDCl_3_, 0.03 wt % TMS) δ 5.38–4.99 (m, 155H), 4.50–4.27 (m, 5H), 1.92 (s, 6H), 1.82–1.34 (m, 499H).

Molecular weight distributions were analyzed on a Shimadzu GPC system equipped with a guard column (KD-G) and analytical column (KD-804) with a target molecular weight range of 2–200 kDa. The mobile phase consisted of HPLC-grade N,N’-dimethylformamide containing 10 mM lithium bromide at a flow rate of 1 mL/min, with the column, UV, and RI detector temperatures maintained at 50 °C. GPC samples were prepared as 2 mg/mL polymer solutions in mobile phase, sonicated for 10 min, and filtered through 0.45 μm PTFE syringe filters prior to injection (100 μL). Molecular weights were determined relative to narrow poly­(methyl methacrylate) standards (Shodex M-75). Polymer size distributions can be found in the SI (SI Figure S1).

#### 2.2.2 Assembly of Mixers

A 3D-printed dual-inlet vortex mixer (DIVM) was employed for the initial mixing step. The mixer was designed using Autodesk Fusion360 and fabricated on a Stratasys Objet30 Pro using Veroclear resin. Postprinting processing involved the removal of support material under running water, installation of threaded heat inserts using an arbor press, and thorough compressed air cleaning to eliminate residual material. O-rings were positioned into the mixing stages, which were then assembled and secured using shoulder bolts, washers, and nuts.

For particle synthesis using the DIVM-MIVM configuration, the outlet of the 3D-printed mixer was connected to a stainless-steel multi-inlet vortex mixer (MIVM) via 0.04″ or 0.06″ inner diameter tubing. For the CIJM-MIVM configuration, a confined impinging jet mixer (CIJM) was connected to an MIVM.

#### 2.2.3 Microparticle Synthesis

MPs were synthesized via Sequential Nanoprecipitation (SNaP) using the described mixer configurations. Before and after the MP synthesis, all mixer streams were flushed with 3 mL of THF (60 mL/min) followed by 3 mL of water (60 mL/min).

For the synthesis of MPs, a THF stream with dissolved PLA and rubrene (2 wt %) was mixed against an equal volume stream of ultrapure water in the first mixer, which was either a 3D-printed DIVM or a CIJM. The output of this first mixing stage flowed into the inlet of the second mixer (MIVM), where it was mixed against three streams of 1 mg/mL PVA in ultrapure water. For the synthesis of itraconazole loaded MPs, itraconazole was added to the THF stream at a 10 wt % total solids concentration.

To achieve the desired *T*_d_, the flow rate for each stream was held constant at 60 mg/mL using a syringe pump (PHD Ultra Syringe pump, Harvard Apparatus) and the length/diameter of the tubing between mixing stages was varied. Stream concentrations, mixer setups and *T*_d_ calculations can be found in the SI (Figures S2 and S3).

To simulate a continuous flow system, MP samples were collected only after the flow had fully developed. This solution was collected in an ultrapure water quenching bath to reduce the final organic concentration to 5 vol % THF. Startup and end volumes were not collected.

MPs were purified via dialysis to remove organic solvent and unencapsulated drug. Using regenerated cellulose dialysis tubing (12–14 kDa MWCO, Repligen), particles were dialyzed against a large excess of ultrapure water, changing the water bath each hour for 6 h.

#### 2.2.4 Particle Characterization

MP morphology was evaluated using scanning electron microscopy (SEM). Samples were prepared by concentrating MPs via centrifugation (2,000 rcf, 5 min) followed by dropwise deposition onto silicon wafers. After ambient drying, samples were sputter-coated with a thin conductive platinum layer (3 nm) using an EMS 150R ES sputter coater and imaged using the SE2 detector on a Zeiss Merlin FE-SEM microscope operating at 1 kV EHT and 110 pA.

Particle size distributions were determined through image analysis of multiple SEM micrographs acquired at 1000× magnification. A custom MATLAB-based circular Hough transform algorithm was employed to detect particles and measure their diameters. For each formulation, a minimum of 2,000 particles were analyzed across multiple fields of view to ensure statistical robustness. Further details can be found in the results section.

Total solids concentration (C_total_) in purified MP suspensions was determined using thermogravimetric analysis (TGA, Discovery 550 TGA, TA Instruments). Samples were heated under nitrogen from 25 to 105 °C at 10 °C/min and maintained at 105 °C for 20 min to ensure complete water evaporation. Total particle concentration was calculated by dividing the residual solid weight by the initial sample volume.

Itraconazole mass concentration (C_drug_) for purified MP samples was determined via High Performance Liquid Chromatography on a Thermofisher Vanquish Core HPLC equipped with an autosampler, quarternary pump, UV detector, and column (Hypersil Gold C18, 3 μm particle size, 175 Å pore size, 4.6 mm × 150 mm, 30 °C). HPLC samples were prepared by diluting NP solutions into acetonitrile to a final 50 vol % acetonitrile. The mobile phase was 50 vol % acetonitrile with 0.1 vol % trifluoracetic acid and 50 vol % water with 0.1 vol % trifluoracetic acid, with a total flow rate of 0.5 mL/min for 20 min. Detection was performed at 256 nm.

MP drug loading wt % (DL) was calculated using the equation $DL=\frac{C_{drug}}{C_{total}}\times 100$ . Encapsulation efficiency (EE%), or the percent of drug that incorporated into MPs, was calculated using the equation $EE\%=\frac{DL}{{DL}_{target}}\times 100$ where DL_target_ was 10% for all batches.

To characterize the itraconazole release rate, MP dispersions were gently concentrated via centrifugation at 2000 rcf for 5 min, followed by decanting off the supernatant. MPs were then redispersed at a 10-fold dilution into sink condition release media comprised of phosphate buffered saline and 10 v/v% Tween-80 surfactant. After initial sampling to determine the initial drug concentration (C_initial_), the dispersion was agitated at 90 rpm in a 37° water bath. At predetermined time points, 1 mL aliquots were withdrawn and centrifuged (2000 rcf, 5 min) to separate MPs from released drug. The supernatant was analyzed via HPLC for released itraconazole (C_released_), with the cumulative release calculated as $\frac{C_{initial}}{C_{released}}\times 100$ for each time point.

## 3 Results and Discussion

In this study, we aimed to identify critical SNaP process and formulation variables and define their optimal operating ranges for robust and tunable MP synthesis. First, we developed a quantitative size analysis methodology for MP size characterization, our key quality attribute of interest. We then investigated the importance of mixing geometry by comparing confined impinging jet mixing and vortex mixing in the first micromixing step which initiates core assembly. Next, we explored the variable workspace of *T*_d_ and C_core_, investigating the influence of each parameter on particle size and identifying regions of predictable particle assembly. Finally, we demonstrated the versatility of SNaP by varying stabilizer composition and encapsulating a model therapeutic agent.

### 3.1 Image Analysis Methodology for Microparticle Characterization

MP size is a key quality attribute that influences particle biodistribution, drug release rate, and efficacy. MPs synthesized in this study exhibited diameters exceeding 1 μm with sedimentation rates on the order of minutes, precluding reliable characterization via conventional dynamic light scattering (DLS) techniques. To address this limitation, we opted to use scanning electron microscopy (SEM) to visualize the MPs. We then developed a high-throughput image analysis protocol capable of providing both quantitative size distribution data and qualitative morphological insights that were critical for evaluating this nascent fabrication process.

Scanning electron microscopy (SEM) was selected as the primary imaging modality due to its superior resolution and magnification capabilities. Upon visual inspection, the SNaP-produced particles consistently exhibited high sphericity, enabling the implementation of a circular Hough transform algorithm for automated particle detection and measurement via a MATLAB script. This image analysis approach successfully detected spherical particles even when partially obscured, permitting accurate analysis of densely populated fields containing thousands of closely packed MPs (Figure 2A-B).

**Figure 2:**
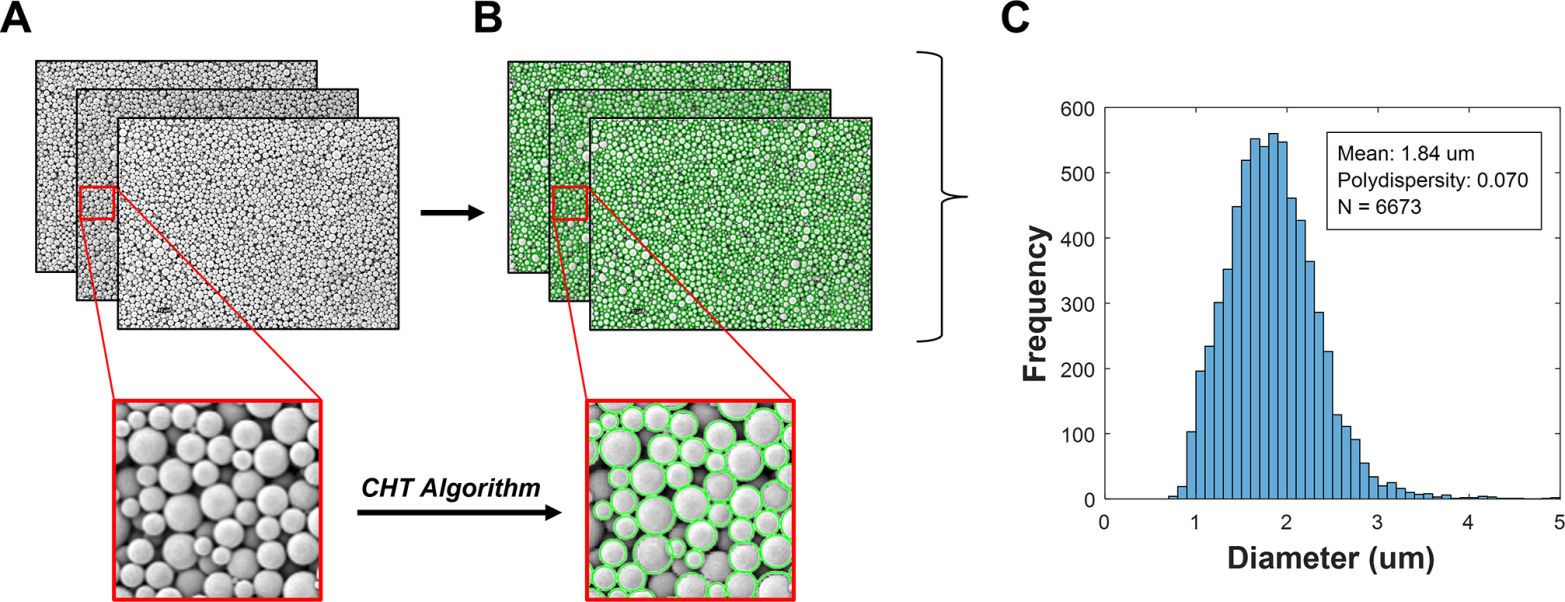
**Image analysis method for characterizing MPs. A.** Raw SEM images and zoomed view. **B.** SEM images with detected circles outlined in green. **C.** Size distribution of pooled diameter data from several images of a MP batch.

To ensure statistical significance, multiple SEM micrographs were captured at 1000× magnification (1024 × 768 resolution) for each formulation. Particle diameters detected via the MATLAB-implemented algorithm were converted to micron scale and aggregated to generate comprehensive size distribution profiles (Figure 2C). Each formulation was independently synthesized in triplicate (N = 3) to evaluate process reproducibility, with a minimum of 2,000 particles analyzed per formulation to ensure robust statistical representation. The microparticle polydispersity index (PDI), the relative variance of particle size assuming a Gaussian distribution, was calculated for each sample as $\frac{\sigma^{2}}{D^{2}}$ , where *D* was mean particle diameter and σ was distribution standard deviation.^25^

### 3.3 Role of Mixing Geometry on SNaP Process

Micromixing efficiency is critical for uniform particle assembly. This is especially true in the first micromixing step that initiates the assembly of the particle core. In previous work, both CIJM and DIVM geometries have been used for the first SNaP micromixing step. We hypothesized that increasing polymer concentrations in the organic stream of the first mixing step (C_core_) would significantly increase solution viscosity, potentially compromising mixing efficiency in less robust mixing configurations. Because vortex mixing has demonstrated superior efficiency compared to confined impinging jet mixing, we compared SNaP MP assembly between these two geometries.^26,27^

We evaluated the CIJM-MIVM and DIVM-MIVM SNaP configurations (Figure 3A and 3D) at fixed *T*_d_ (30 ms) and explored the C_core_ concentrations, 40 and 100 mg/mL PLA, which represent the low and high extremes of the concentration range. MPs were stabilized by 0.1 wt % PVA in all three water streams in the second mixing step. The organic stream dynamic viscosities were calculated for C_core_ of 40 and 100 mg/mL using the Mark–Houwink-Sakurada equation for intrinsic viscosity [η] (eq 1) and a modified Huggins equation for solution viscosity η (eq 2):^28,29^

$$\left[\eta \right]=KM^{a}$$ 1

$$\eta =\eta_{s}\left(1+\left[\eta \right]c+k_{H}{\left[\eta \right]}^{2}c^{2}\right)$$ 2

where K and a represent Mark–Houwink parameters for PLA in THF (0.0174 mL/g and 0.736, respectively^28^), M is the polymer molecular weight (17.5 kDa), η_s_ is THF viscosity (0.46 mPa·s), and k_H_ is the Huggins coefficient (0.3 for good solvents). This analysis revealed a greater than 2-fold increase in solution viscosity between 40 mg/mL (0.99 mPa•s) and 100 mg/mL (2.21 mPa•s) formulations. Full calculation details can be found in the SI (Figure S4).

**Figure 3:**
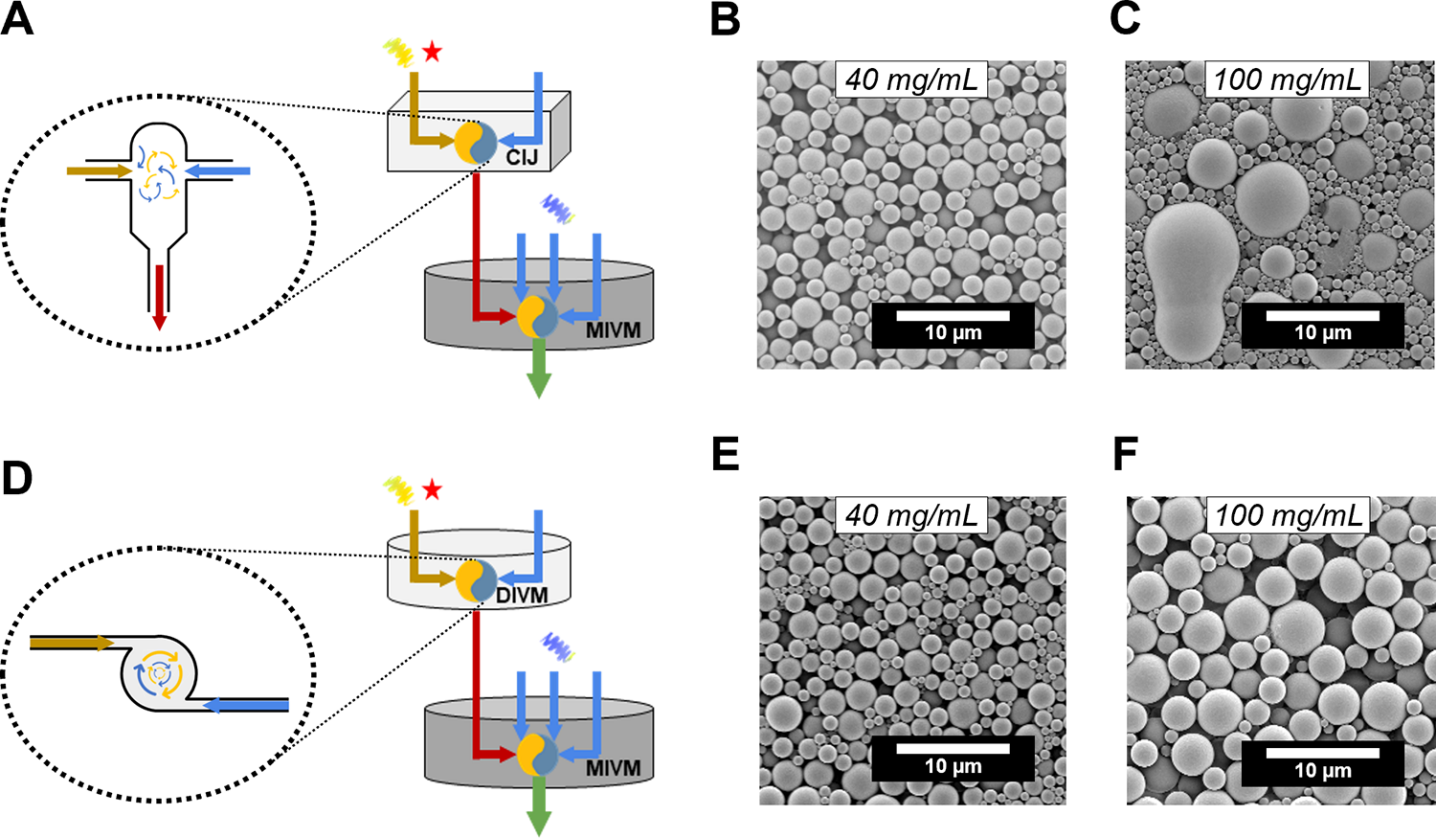
**Mixing geometry influences operating variable space. A.** Mixer setup schematics and mixing geometries for the CIJM-MIVM. **B,C**. Representative images of MPs synthesized at the labeled C_core_ for the CIJM-MIVM. **D.** Mixer setup schematics and mixing geometries for the DIVM-MIVM **E,F.** Representative images of MPs synthesized at the labeled C_core_ for the DIVM-MIVM configurations.

In the CIJM-MIVM setup, uniform particle assembly was observed at 40 mg/mL, producing monodisperse 1.9 μm particles (Figure 3B). However, at 100 mg/mL, severe polydispersity and irregular morphologies emerged, including pear and disc-shaped aggregates—indicators of insufficient mixing during core assembly (Figure 3C). In contrast, the DIVM-MIVM configuration maintained uniform assembly at both concentrations, producing spherical populations with diameters of 1.6 and 2.2 μm, respectively (Figures 3E-F). These observations were consistent at a longer delay times (*T*_d_ = 90 ms), where the CIJM-MIVM configuration produced 3.0 μm particles at 40 mg/mL, and aggregates at 100 mg/mL, and the DIVIM-MIVM configuration produced 2.2 and 3.0 μm particles at the same respective C_core_ values (SI Figure S5).

The superior performance of the vortex mixing geometry likely stems from its enhanced tolerance to increased stream viscosity, effectively expanding the operational parameter space for C_core_ conditions. Notably, vortex mixing accommodates asymmetric flow rates, enabling greater flexibility in antisolvent/solvent ratios and supersaturation states. Based on these advantages, the DIVM-MIVM configuration was selected for subsequent investigations.

### 3.4 Controlling Microparticle Sizes via Delay Time and Core Concentration

Having established the optimal mixing configuration, we proceeded to investigate the primary parameters controlling MP formation during SNaP. Our previous study identified *T*_d_, the residence time between core nucleation in the first mixer and stabilization in the second mixer, as a critical determinant of MP size.^18^ While our earlier work demonstrated size-tunable assembly of MPs from 0.7 to 1.2 μm using delay times of 7–23 ms,^18^ the present study extends this investigation to longer delay times to access larger MP size regimes.

To achieve the extended delay times, we connected two millifluidic mixers in series with interchangeable tubing with variable dimensions. The system consisted of a DIVM for the imitation of core formation followed by an MIVM for stabilization. The delay time between mixing steps was controlled by adjusting the thickness and length of the connecting tubing between micromixers. At constant flow rate *Q*, the delay time was calculated using eqs 3 and 4:

$$V=\left(V^{\prime }+a^{*}L\right)$$ 3

$$T_{d}=\frac{V}{\sum Q_{i}}$$ 4

where *V* represents the total delay channel volume, *a* and *L* denote the cross-sectional area and length of the interchangeable tubing, and *V’* accounts for the volume contribution of fixed delay channel segments. By maintaining a constant flow rate of 60 mL/min in each inlet while varying the tubing dimensions, we established delay times of 30, 60, and 90 ms (Figure 4).

**Figure 4:**
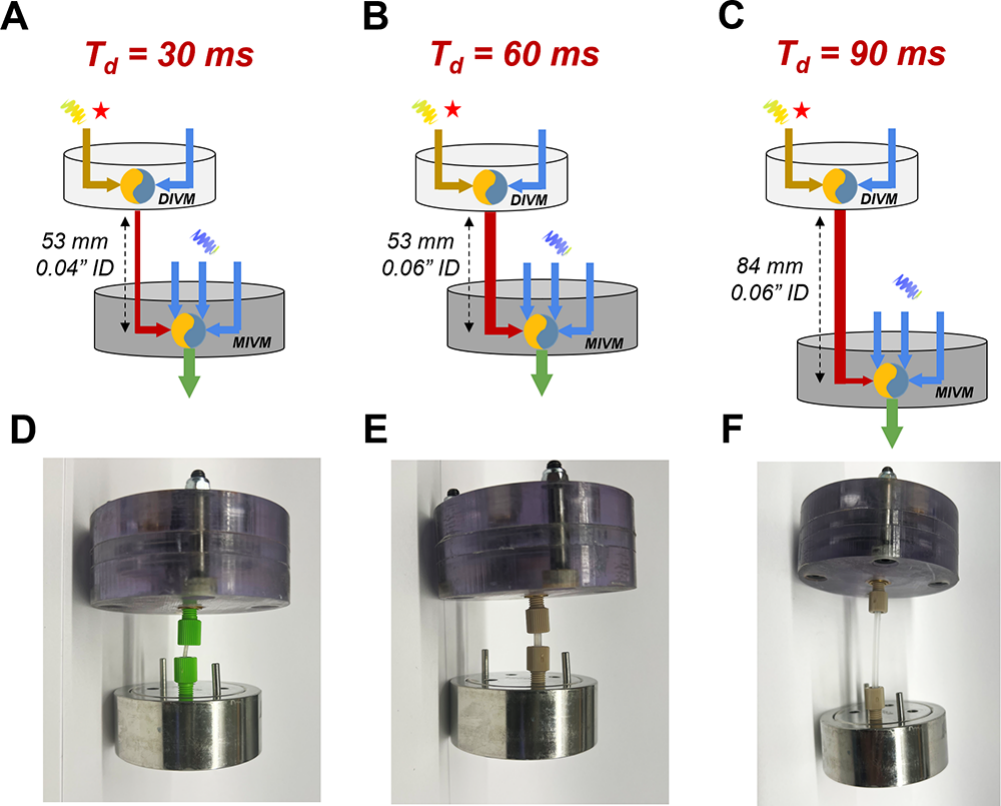
**A-C.** DIVM-MIVM setup schematics for *T*_d_ of 30, 60, and 90 ms, respectively. **D-F.** Respective photos of each setup, showing replaced delay tubing thickness and length.

Concurrently, we investigated the influence of C_core_ on MP characteristics. Previous FNP studies have demonstrated that increasing the total solids concentration results in larger nanoparticles for systems involving core material aggregation.^20,21,30^ We hypothesized that similar trends would occur with C_core_ concentrations in the SNaP process, giving us a facile lever for tuning MP size. We evaluated C_core_ values ranging from 40 to 100 mg/mL PLA, with the upper bound approaching the solubility limit of PLA (18 kDa) in THF, which we determined experimentally to be approximately 120 mg/mL.

Within these parameter ranges, we mapped the operational space for SNaP by determining achievable MP sizes (Figure 5A). As expected, increasing *T*_d_ led to larger MP sizes across all core concentrations, though the effect was less pronounced at the 100 mg/mL PLA C_core._ This is consistent with our hypothesis that core growth continues during the *T*_d_ between two mixers. Longer core assembly times therefore result in larger particles. Similarly, increasing C_core_ produced larger particles, giving us an additional lever for controlling MP size. This effect likely stems from an increase in the core growth rate relative to the core nucleation rate, similar to single step nanoprecipitation.^30^ Within this variable space, we were able to produce MPs ranging from 1.59 ± 0.01 μm (40 mg/mL PLA, 30 ms *T*_d_) to 2.98 ± 0.35 μm (100 mg/mL PLA, 90 ms *T*_d_) with narrow size distributions. It should be noted that the MP PDIs did increase at the higher 80 and 100 mg/mL PLA C_core_ conditions. Example SEM images of the spherical MPs are shown in Figure 5D-F. The complete data set can be found in Table 1.

**Figure 5:**
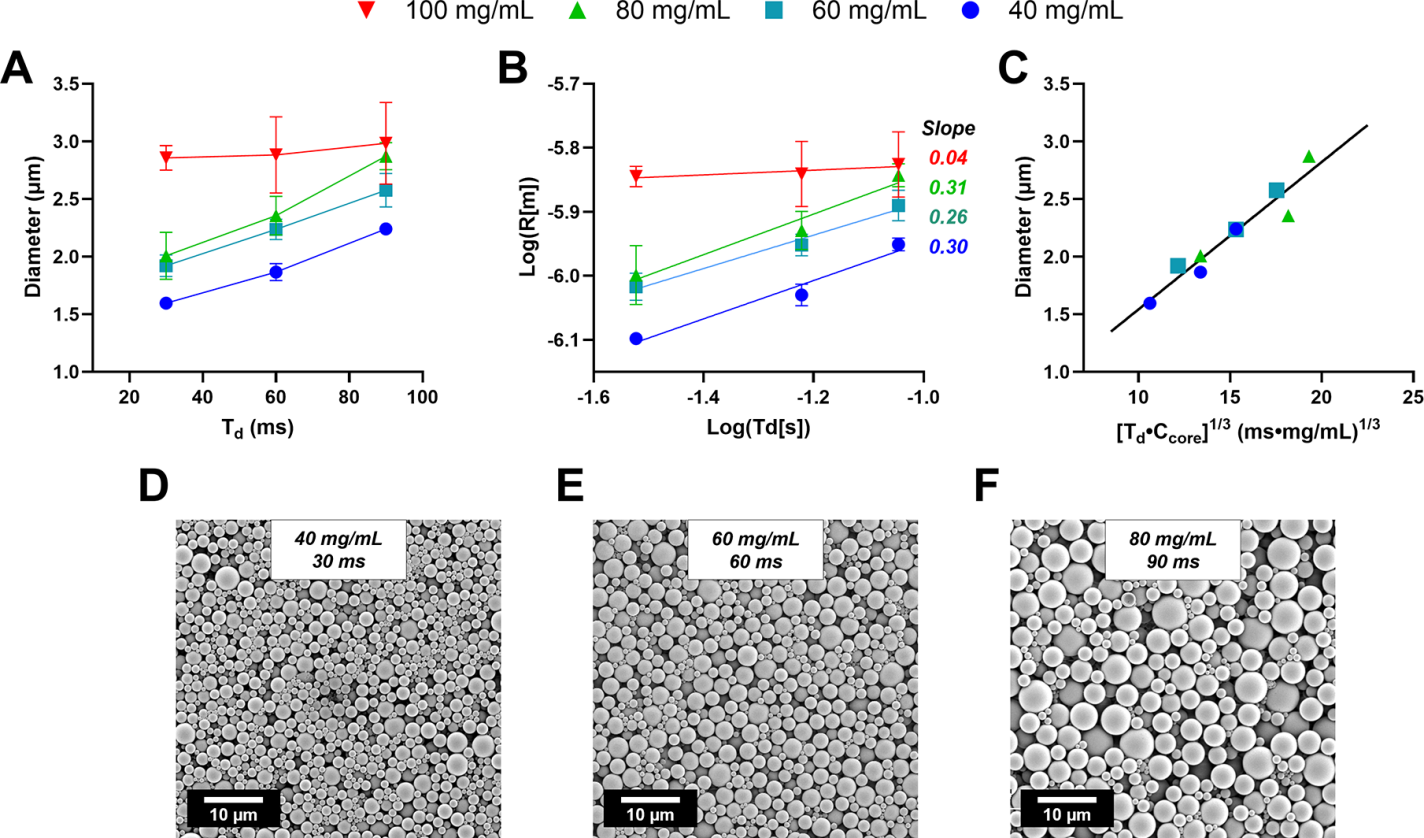
**Influence of** *T*_**d**_ **and C**_**Core**_ **on microparticle size. A.** Particle diameter as a function of *T*_d_ for each C_core_. Influence of both *T*_d_ and C_core_ on diameter were calculated to be statistically significant by 2way ANOVA test (α = 0.5, *P* < 0.0001 for both parameters). **B.** Linearized radius vs *T*_d_, plotted on a logarithmic scale. **C.** Particle diameter as a function of the products of *T*_d_ and C_core_ scaled to 1/3, with the linear relationship of C_core_ 40, 60, and 80 mg/mL plotted. **C-E.** Representative images of MP batches at labeled conditions.

**Table 1:** Average MP Size and PDI from Organic Stream Concentration (C_core_) and Delay Times (*T*_d_) Tested (N = 3)^a^

***C*_*core*_** (mg/mL)	***T*_*d*_ *(ms)***	***diameter (*** **μ*m)***	***PDI***
40	30	1.59 ± 0.01	0.28 ± 0.02
40	60	1.86 ± 0.07	0.12 ± 0.01
40	90	2.24 ± 0.05	0.15 ± 0.05
60	30	1.92 ± 0.09	0.11 ± 0.03
60	60	2.24 ± 0.09	0.17 ± 0.04
60	90	2.57 ± 0.15	0.12 ± 0.05
80	30	2.01 ± 0.20	0.29 ± 0.15
80	60	2.36 ± 0.17	0.27 ± 0.10
80	90	2.87 ± 0.12	0.34 ± 0.09
100	30	2.86 ± 0.11	0.17 ± 0.03
100	60	2.88 ± 0.33	0.24 ± 0.11
100	90	2.98 ± 0.35	0.39 ± 0.15

a Dissolved solids for all formulations contained 2 wt % rubrene, e.g. core concentration 40 mg/mL corresponds to 39.2 mg/mL PLA and 0.8 mg/mL rubrene. (*N* = 3).

Previously, we have used Smoluchowski diffusion-limited growth kinetics to understand nanoparticle and microparticle assembly via SNaP.^18^ For delay times below 23 ms, we demonstrated that the particle radius, R, scaled with *T*_d_ as described by Smoluchowski’s model of diffusion limited growth shown in eq 5:

$$R={\left(\frac{tk_{B}TC_{core}}{\pi \eta \rho_{core}}\right)}^{1/3}$$ 5

where t is growth time (in our case *T*_d_), T is temperature, η is solution dynamic viscosity, ρ is core material density, C_core_ is the core concentration, and k_B_ is the Boltzmann constant.^18^ This supported our hypothesis that SNaP particle formation proceeds via diffusion-limited aggregation of hydrophobic species in the delay channel.^18^ In the current study, we extended this analysis to longer delay times up to 90 ms. When linearizing our microparticle size data (Figure 5B), we observed two distinct trends depending on core concentration. For C_core_ values of 40, 60, and 80 mg/mL, the data followed diffusion-limited growth kinetics with slope values of approximately 1/3, consistent with Smoluchowski’s model. However, at 100 mg/mL C_core_, this relationship broke down, and particle size became largely independent of delay time, suggesting a shift in the underlying assembly mechanism.

We hypothesized that at 100 mg/mL PLA, we were approaching the critical overlap concentration (C*) where PLA chains begin to entangle in solution and thus no longer precipitate as individual globules prior to assembly. To test this hypothesis, we calculated C* for our linear PLA (17.5 kDa) by first determining the radius of gyration (*R*_g_) using De Gennes’s scaling law (eq 6)^31^ and then calculating the C* using coil overlap concentration eq (eq 7):^32^

$$R_{g}^{2}=\frac{Nb^{2}}{6}$$ 6

$$C^{*}≃\frac{3M}{4\pi N_{A}{R_{g}}^{3}}$$ 7

where N is the number of Kuhn segments (121), b is the Kuhn length (8.81 Å), M is molecular weight (17.5 kDa), and N_A_ is Avogadro’s number.^33^ Kuhn length calculations can be found in the SI. The calculated *R*_g_ value of 39.6 Å yielded a C* of 112 mg/mL, confirming that our 100 mg/mL formulation approached the threshold for chain entanglement. This explains the deviation from Smoluchowski kinetics at this concentration, as polymer chain entanglements can lead to network formation during precipitation, altering the fundamental assembly mechanism. Additional experiments at a C_core_ of 120 mg/mL (above C*) resulted in visible PLA macroprecipitates immediately after synthesis, further confirming that polymer chain entanglements in the organic stream lead to uncontrolled aggregation. These findings establish an important process constraint: polymeric materials in SNaP should remain below their overlap concentration in the solvent stream to ensure controlled assembly.

Next, using the Smoluchowski scaling law, we successfully collapsed MP size data from formulations with 40, 60, and 80 mg/mL PLA C_core_’s onto a single predictive curve (Figure 5C). This notable finding demonstrates that particle size in SNaP processes follows fundamental diffusion-limited aggregation principles, enabling precise prediction of microparticle dimensions based on C_core_ and *T*_d_. Such predictive capability represents a significant advancement in controlled microparticle manufacturing.

Based on our comprehensive analysis, we developed a process phase diagram for 17.5 kDa PLA microparticles (Figure 6) that delineates regions of controlled, diffusion-limited growth from zones of process instability. This diagram serves as a practical guide for formulation scientists, enabling rational selection of formulation (C_core_) and process parameters (*T*_d_) to achieve target particle specifications. Within the stable operating region, we demonstrated reproducible size control from 1.6 to 2.9 μm by systematically varying core concentration and delay time.

**Figure 6:**
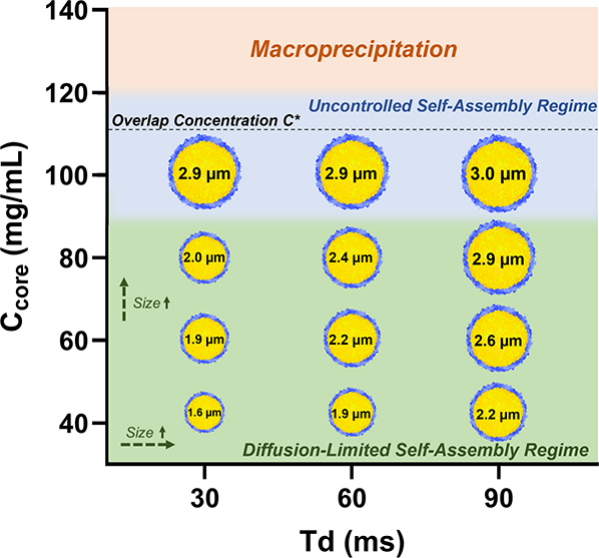
**SNaP process phase diagram for PLA microparticles.** Summarized results of MP size tuning via C_core_ and *T*_d_. Relative particle sizes are illustrated with process instability zones shaded.

The industrial relevance of this approach is underscored by the ability to work with small batches for formulation development or to scale up under continuous flow. For a single batch, accounting for start-up volume and flow stabilization, we used between 120 and 300 mg of PLA, making this approach material-efficient for early development studies. A single lab-scale SNaP mixer setup operated under continuous flow yields 144 to 360 g of microparticles hourly (18 L/h). Furthermore, the vortex mixing geometries employed in the SNaP mixers are amenable to scale-up.^23,24^

### 3.5 Variation of Stabilizing Polymers

The surface properties of drug delivery vehicles significantly influence their biological behavior. To demonstrate the versatility of SNaP for creating MPs with diverse surface chemistries, we synthesized PLA particles using three different stabilizing polymers: poly­(vinyl alcohol) (PVA, 18 kDa), polyvinylpyrrolidone (PVP, 40 kDa), and the amphiphilic block copolymer polyethylene glycol-polylactic acid (PEG–PLA, 5 kDa-5 kDa). All formulations used identical core streams (C_core_ = 40 mg/mL PLA) and delay times (*T*_d_ = 13.5 ms). The concentrations of PVP and PEG–PLA were chosen based upon concentration ranges used in literature.^34,35^

While hydrophilic stabilizers (PVA and PVP) were introduced in the aqueous streams of the second mixing step, the amphiphilic PEG–PLA was incorporated in a second organic stream that replaced one of the water streams. As shown in Figure 7, all three formulations produced MPs with virtually identical morphologies and mean diameters. PVP-stabilized MPs exhibited slightly higher polydispersity, likely attributable to the substantially higher molecular weight of PVP (40 kDa) compared to PVA (18 kDa) and PEG–PLA (10 kDa), resulting in slower diffusion kinetics during the stabilization phase.

**Figure 7:**
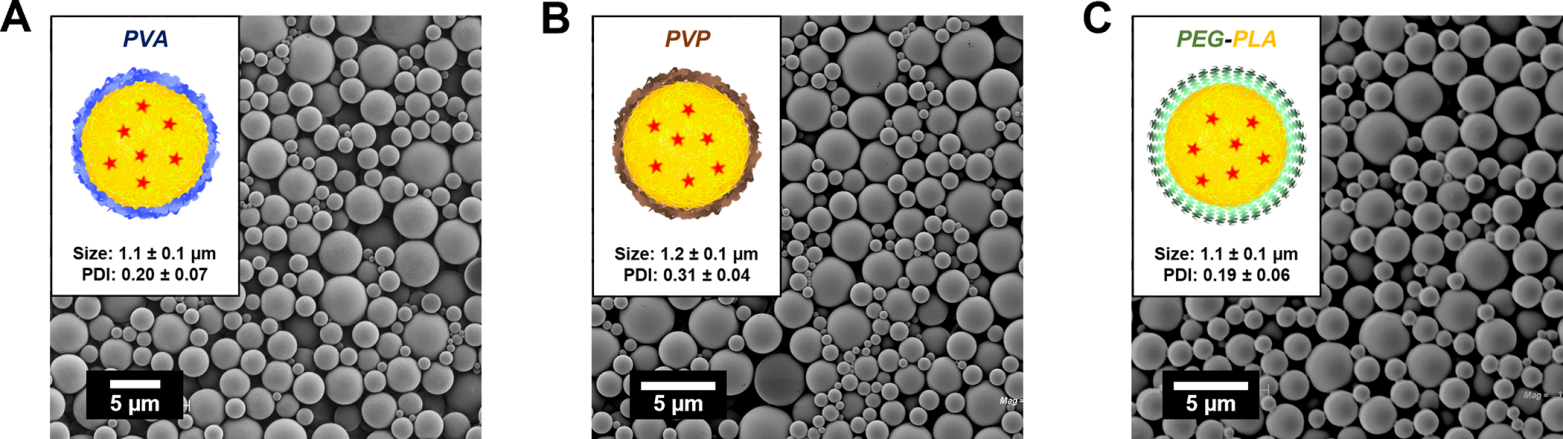
SEM images and annotated sizes of PLA MPs stabilized by **A.** PVA **B.** PVP, and **C.** PEG–PLA. (*N* = 3).

The consistency in particle size across diverse stabilizers with fundamentally different stabilization mechanisms - adsorption of water-soluble polymers versus hydrophobic anchoring of amphiphilic copolymers - provides compelling evidence that MP size is predominantly determined by core growth during the intermixer delay time rather than by stabilizer characteristics. This mechanistic insight further supports the robustness and versatility of the SNaP process for producing MPs with tailored surface properties.

### 3.6 Loading Small Molecule Therapeutics into Microparticles via SNaP

To evaluate the pharmaceutical relevance of SNaP-produced MPs, we investigated the encapsulation of itraconazole, a weakly hydrophobic antifungal agent (Figure 8A). To be a viable process for therapeutic MP production, high encapsulation efficiency of small molecule drugs is imperative. Itraconazole was incorporated into the core organic stream at a 10 wt % target loading while maintaining C_core_ at 60 mg/mL. Delay times were varied from 30 to 90 ms to assess the influence of particle size on drug loading efficiency.

**Figure 8:**
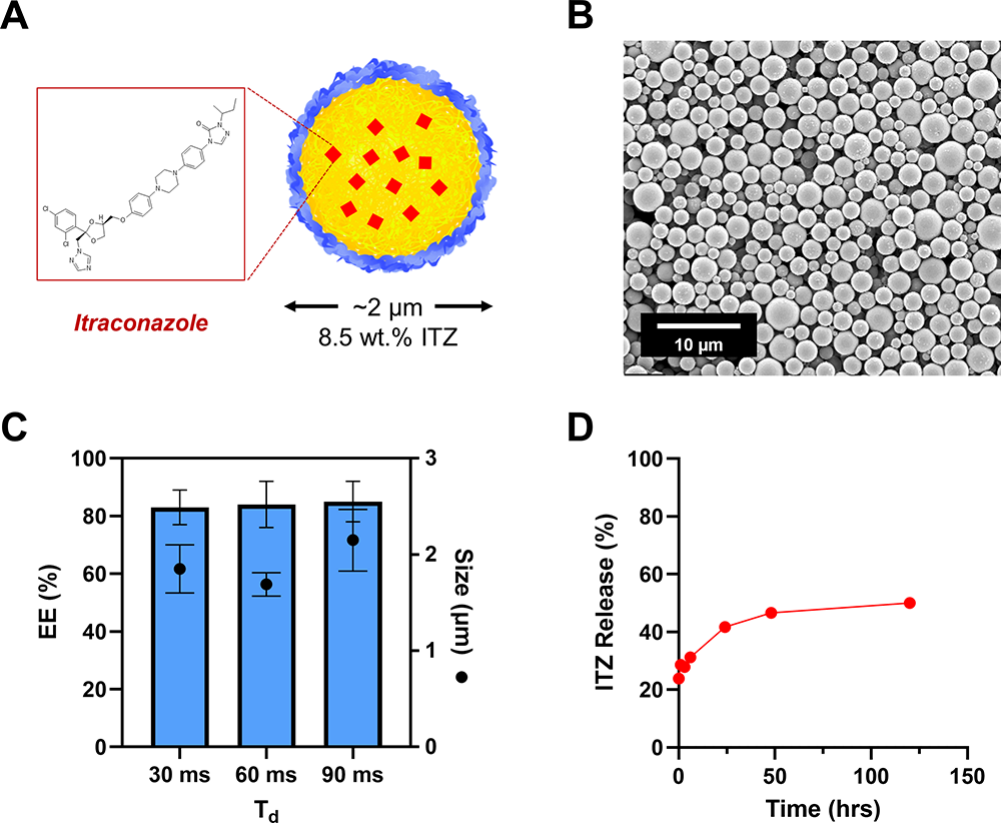
**Characterization of itraconazole-loaded MPs A.** Illustration of itraconazole-loaded SNaP MP, **B.** Representative image and size distribution. **C.** Summarized size and encapsulation efficiencies. **D.** Cumulative itraconazole release from MPs (60 mg/mL, 90 ms) over 5 days in sink conditions at 37 °C (*N* = 3, error bars smaller than symbol height).

Itraconazole loaded MPs were successfully synthesized at all three delay times. A representative SEM image of the spherical MPs is shown in Figure 8B. Shown in Figure 8C, the drug-loaded MPs exhibited slightly smaller diameters than their nondrug-loaded counterparts, attributable to the higher density of itraconazole (1.60 g/mL) compared to PLA (1.25 g/mL). Encapsulation efficiency remained remarkably consistent across all delay times, with values of 83%, 84%, and 85% for 30, 60, and 90 ms, respectively. These efficiencies are comparable to those achieved in small molecule-loaded polymeric MPs synthesized via industrial-scale emulsification techniques,^36^ demonstrating the pharmaceutical relevance of the SNaP process. Moreover, the consistent drug loading efficiency across varying delay times demonstrates that MP size can be independently tuned without compromising therapeutic incorporation, highlighting the orthogonal control of critical quality attributes afforded by the SNaP process.

Release kinetics studies revealed a biphasic pattern characteristic of matrix-type polymeric delivery systems:^37,38^ an initial burst release of approximately 23% upon dispersion into sink conditions, followed by a slow sustained release over the 5 days tested (Figure 8D). This release profile indicates the potential utility of SNaP-produced MPs for extended-release pharmaceutical applications.

## 4 Conclusions

In this work, we have established Sequential NanoPrecipitation (SNaP) as a robust and versatile platform for the controlled assembly of polymeric microparticles. By systematically investigating key process parameters, we have developed a comprehensive understanding of the mechanisms governing particle formation and size control in this nascent technology. Our findings demonstrate that vortex mixing geometries provide superior performance in the core assembly step, particularly at higher polymer concentrations where increased solution viscosity can compromise mixing efficiency in confined impinging jet systems. Through careful manipulation of *T*_d_ and core concentration C_core_, we achieved precise size control of monodisperse microparticles ranging from 1.6 to 3.0 μm - dimensions particularly valuable for applications in pulmonary drug delivery.

The mechanistic insights gained from this study are significant; we confirmed that particle assembly follows Smoluchowski diffusion-limited growth kinetics across a wide operating space, enabling predictive modeling of particle size based on fundamental process parameters. We identified critical process boundaries, particularly the polymer overlap concentration threshold, above which controlled assembly transitions to undesirable aggregation.

The versatility of SNaP was further demonstrated through successful particle formation using diverse stabilizing polymers while maintaining consistent size control, highlighting the process’s adaptability to different surface functionalization strategies. Most importantly, we validated the pharmaceutical relevance of SNaP by achieving consistently high encapsulation efficiency (83–85%) of itraconazole across various formulations, with release profiles suitable for extended-release applications.

SNaP uniquely bridges the gap between laboratory precision and industrial scalability. Our lab-scale system demonstrated production rates of 144 to 360 g of microparticles per hour, with established potential for scale-up using larger mixing geometries. This combination of precise size control, predictable assembly behavior, material efficiency, and scalability positions SNaP as a transformative approach for polymeric microparticle production that overcomes the longstanding dichotomy between laboratory-scale precision and industrial manufacturing requirements.

Future investigations will focus on determining the role of solvent quality in the first mixing step and further expanding the achievable size range by increasing delay times.

## Supplementary Material



## Notes

PLA characterization; SNaP mixer setup images; delay time calculations; Reynolds number calculations; extended CIJM-MIVM and DIVM-MIVM comparison; PLA overlap concentration calculation details; tabulated data; HPLC details (PDF)
