# Supplementary material for: Process and Formulation Parameters Governing Polymeric Microparticle Formation via Sequential NanoPrecipitation (SNaP)
Source: ACS Eng Au. 2025 Jul 4;5(4):468–77. doi: 10.1021/acsengineeringau.5c00035 (PMC12371724; doi:10.1021/acsengineeringau.5c00035)
Supplement: Supplementary file 1 [file eg5c00035_si_001.pdf]

Supplementary Information

Process and Formulation Parameters Governing Polymeric Microparticle Formation via Sequential NanoPrecipitation (SNaP)

Parker K. Lewis[1], Nouha El Amri[1], Erica E. Burnham[1], Natalia Arruz[3], Nathalie M. Pinkerton[1,2*]

[1]Department of Chemical and Biomolecular Engineering, Tandon School of Engineering, New York University, NY, 11201, USA

[2]Department of Biomedical Engineering, Tandon School of Engineering, New York University, NY, 11201, USA

[3]Department of Mechanical and Aerospace Engineering, Tandon School of Engineering, New York University, NY, 11201, USA

* Email: nathalie.pinkerton@nyu.edu

1. List of Abbreviations:

CIJM: Confined Impinging Jet Mixer

C_{core}: Core Concentration

DIVM: Dual-Inlet Vortex Mixer

DL: Drug Loading

EE: Encapsulation Efficiency

FNP: Flash NanoPrecipitation

HPLC: High Performance Liquid Chromatography

MIVM: Multi-Inlet Vortex Mixer

MP: Microparticle

NP: Nanoparticle

PDI: Polydispersity Index

PEG-PLA: Poly(ethylene glycol)–Poly(lactic acid

PLA: Poly(lactic acid)

PVA: Poly(vinyl alcohol)

PVP: Poly(vinyl pyrrolidone)

Re: Reynolds Number

SEM: Scanning Electron Microscopy

SNaP: Sequential NanoPrecipitation

T_d : Delay time

TGA: ThermoGravimetric Analyzer

2. Characterization of Poly(lactic acid)

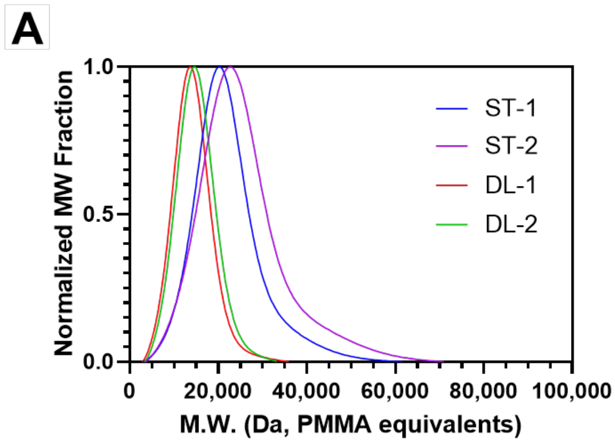

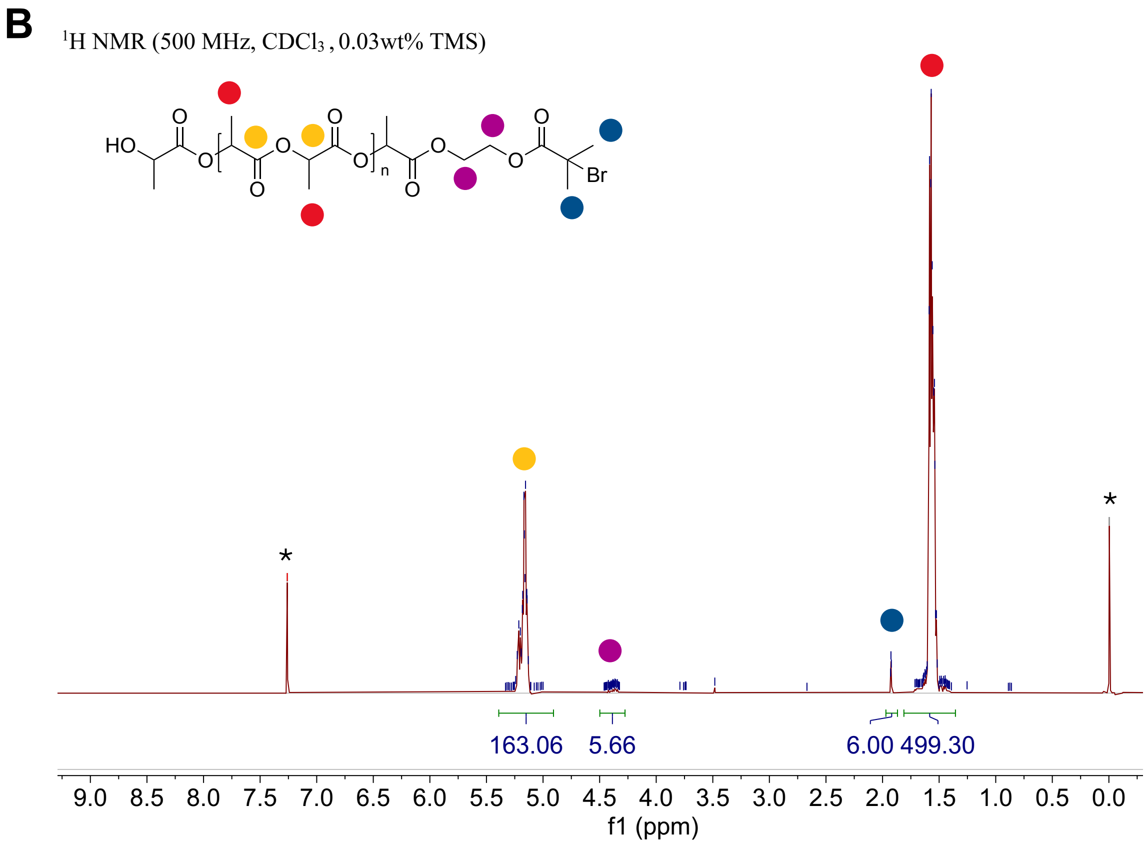

Figure S1. **A.** Molecular weight distributions of in-house PLA from gel permeation chromatography (GPC). “DL” batches were used for itraconazole loaded microparticles, while “ST” batches were used for all other size tuning and mixing investigations. **B.** Representative HNMR spectrum, peaks labeled with respective PLA structure. Peak at 7.26 ppm corresponds to $CDCl_3$ NMR solvent. Peak at 0 ppm corresponds to the TMS standard.

Table S1. Tabulated molecular weights of in-house PLA from GPC and HNMR. M_n and M_w values are reported in terms of polymethyl methacrylate (PMMA) standards or PMMA equivalent molecular weight.

	**1H NMR**	**GPC**		
Sample	***Mn (Da)***	***Mn (Da)***	***Mw (Da)***	***Đ****
ST-1	10,877	17,491	20,160	1.15
ST-2	12,017	19021	22,525	1.18
DL-1	8,691	11,657	13,165	1.13
DL-2	8,643	12,572	14,053	1.12

**Đ* is the polydispersity index, M_w/M_n

3. SNaP Mixer Set-Up

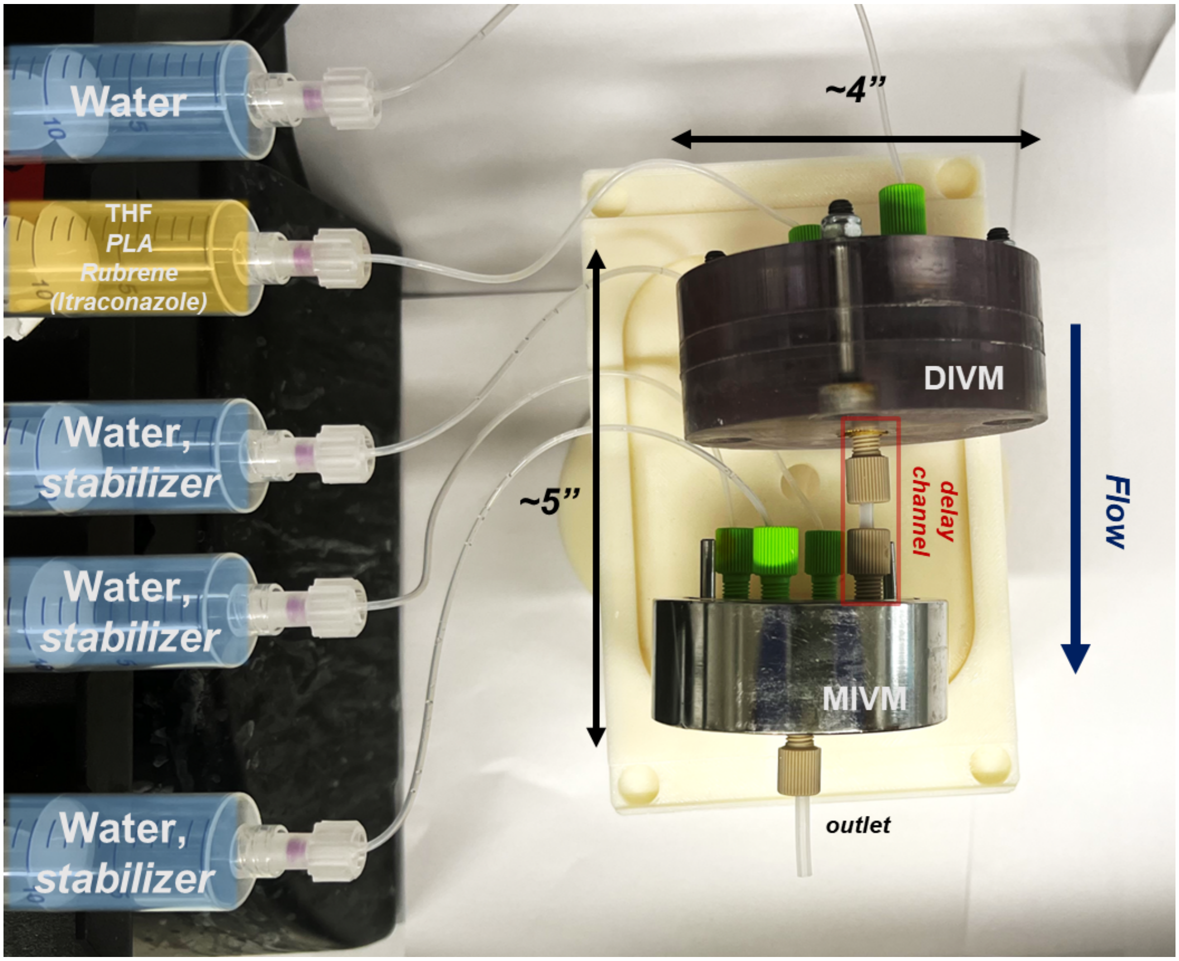

Figure S2. Annotated photo of DIVM-MIVM SNaP Mixer configuration (60 ms) with syringe pump setup.

4. Delay Time (T_d) Calculations

$$(1)\ \boldsymbol{V} = (\boldsymbol{V'} + \boldsymbol{a} * \boldsymbol{L})$$
$$(2)\ \boldsymbol{T_d} = \frac{\boldsymbol{V}}{\sum \boldsymbol{Q_i}}$$

V = tunable residence volume of delay channel intermediate tubing

V' = non-tunable residence volume of mixer delay channels (mixer 1 outlet channel, mixer 2 inlet channel)

a = cross sectional area of delay channel intermediate tubing

L = tunable length delay channel intermediate tubing

T_d = delay time (residence time from first mixing geometry to second mixing geometry)

Q_i = volumetric flow rate of each incoming stream to first mixing geometry

Equations S1 and S2. S1) Calculation of residence volume from the outlet of the first mixing geometry to the entrance of the second mixing geometry. S2) calculation of residence time, or delay time, in this channel, corresponding to the timescale of the core growth stage.

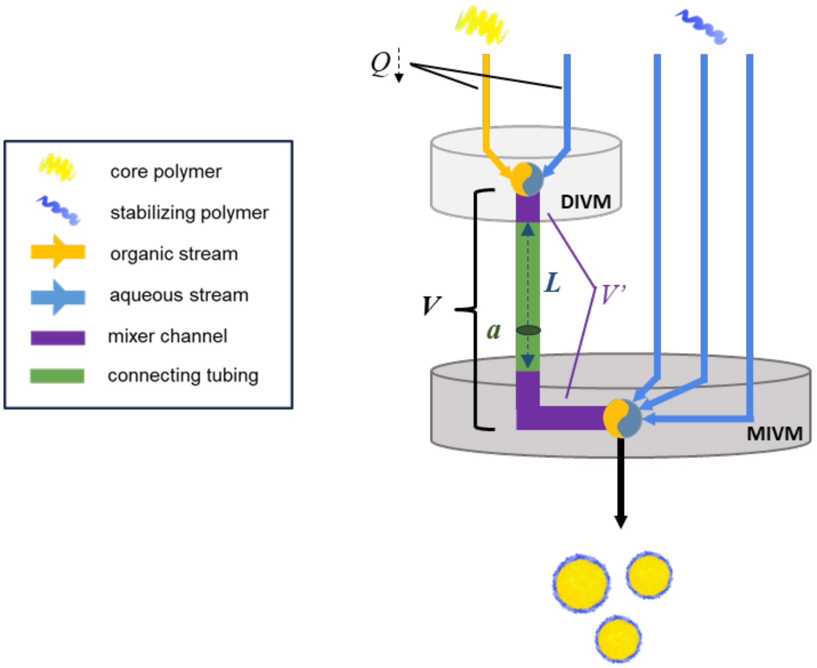

Figure S3. SNaP setup schematic showing delay time calculation parameters.

Table S2. Characteristic 3D printed SNaP mixer properties and calculated delay times from equations S1 and S2 for configurations used in Figures 2D and 4D.

T_d (ms)	***L (mm)***	***a (m^2)***	***V (m^3)***	***Q (mL/min)***
30	53	8.11E-07	6.02E-08	60
60	53	1.94E-06	1.20E-08	60
90	84	1.94E-06	1.80E-08	60

All inlet streams in this study were flowed at the same rate, so Q was equivalent for each stream. L and D correspond to the length and inner diameter of interchangeable tubing in between vortex mixers. V' was calculated using dimensions from original schematics of the DIVM and technical drawings of the MIVM. $V' = 1.73 \times 10^{-8}\ m^3$

5. Calculation of Mixing Chamber Reynolds Numbers

Equation S3 describes the calculation of velocity of a single stream leading into a mixing geometry and S4 describes the calculation for the Reynolds number in a mixing geometry with *i* incoming streams.[1]

(3) $\boldsymbol{U_i} = \frac{\boldsymbol{Q_i}}{\boldsymbol{a}}$

(4) $\boldsymbol{Re} = \sum_{\boldsymbol{i=1,N}} \frac{\boldsymbol{U_i}}{\boldsymbol{v_i}} \boldsymbol{D}$

U_i = average velocity of incoming mixing stream i

Q_i = volumetric flow rate of incoming mixing stream i

a_1 = cross sectional area of DIVM incoming mixing channels

a_2 = cross sectional area of MIVM incoming mixing channels

D = vortex mixing geometry diameter

Re = Reynold's number

v_i = kinematic viscosity of incoming mixing stream i

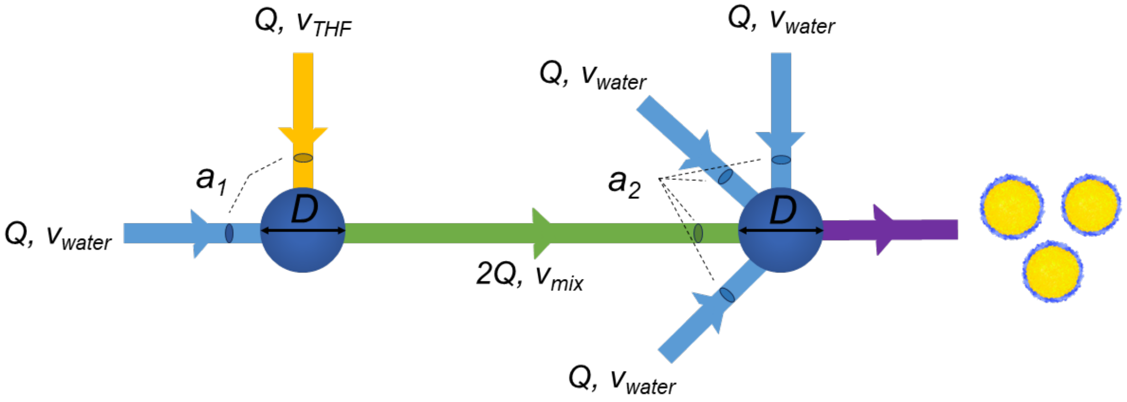

Figure S4. Flow path schematics of SNaP mixer setup and relevant Reynolds number parameters.

Re_1 is the Reynolds number in the first mixing geometry, and Re_2 is the Reynolds number in the second mixing geometry.

$a_1 = 5.63 \times 10^{-7}\ m^2$

$a_2 = 5.81 \times 10^{-7}\ m^2$

$v_{water} = 8.92 \times 10^{-7}\ m^2/s$

D = 0.005 m

Kinematic viscosity was calculated for each C_{core}, as dissolved PLA resulted in a significant increase in solvent stream viscosity. Contribution of PLA to the overall organic stream viscosity (intrinsic viscosity) was first calculated using the Mark-Houwink equation (S5) for intrinsic viscosity $[\eta]$ of a polymer, where K and a are the Mark-Houwink parameters for PLA in THF, with values of 0.0174 mL/g and 0.736, respectively, and M is the molecular weight (17.5 kDa).[2] Intrinsic viscosity $[\eta]$ of the PLA used in THF was calculated to be 23.1 mL/g.

Equation 2 calculates kinematic viscosity v from solvent viscosity η_s, intrinsic viscosity $[\eta]$, and solution density ρ using a modified Huggins equation (S6), where the Huggins coefficient k_H is assumed to be 0.3 due to the high solubility of PLA in THF.[3] Here, η_s is the dynamic viscosity of pure THF, 0.46 mPa*s. The density of each organic stream was found by adding the respective total solids concentration to the density of THF (0.888 g/mL).[4]

$$(5)\ [\eta] = KM^a$$
$$(6)\ v = \frac{\eta_s}{\rho}(1 + [\eta]c + k_H[\eta]^2c^2)$$

The above calculations yield the following values for stream densities, viscosities (dynamic and kinematic), and Reynolds numbers in the first mixing step in the DIVM-MIVM SNaP configuration.

Table S3. Reynolds number calculation parameters for the first mixing step where the organic stream (org) mixes against one stream of water.

C_{core} *(mg/mL)*	ρ_{org} *(g/mL)*	η_{org} *(g/cm*s)*	v_{org} *(m²/s)*	Re_1
40	0.928	0.0099	1.07E-06	18308
60	0.948	0.0134	1.41E-06	16264
80	0.968	0.0174	1.80E-06	14901
100	0.988	0.0221	2.23E-06	13948

For incoming streams to the second mixing step, dynamic viscosity for 50/50 v/v% water/THF mixture in the delay channel was calculated using the Arrhenius rule for liquid mixtures:[5]

$$(7)\ \ln(\eta_{mix}) = \sum_{i=1}^{N} x_i ln\eta_i$$

η_{mix} = dynamic viscosity of liquid mixture

x_i = mole fraction of mixture component i

The values x_{water} and x_{THF} for a 50/50 v/v% mixture were calculated to be 0.802 and 0.198, respectively based on the molar volumes of water (18.0 mL/mol) and THF (72.8 mL/mol). Molar contributions of dissolved solids in the THF stream were not considered in this calculation as they make up less than 0.01% of total moles in the organic stream. Final kinematic viscosity v_{mix} of this mixed stream was found by dividing dynamic viscosity by the averaged mass densities of water and the respective organic stream.

Table S4. Reynolds number calculation parameters for the second mixing step, where the first mixing step output (50 v/v% water, 50 v/v% original organic stream) mixes against three additional streams of water.

C_{core} (mg/mL)	ρ_{mix} (g/mL)	η_{mix} (g/cm*s)	v_{mix} (m^2/s)	Re_2
40	0.964	0.0100	1.04E-06	45588
60	0.974	0.0106	1.09E-06	44782
80	0.984	0.0112	1.14E-06	44127
100	0.994	0.0117	1.18E-06	43584

6. CIJM-MIVM vs DIVM-MIVM Comparison

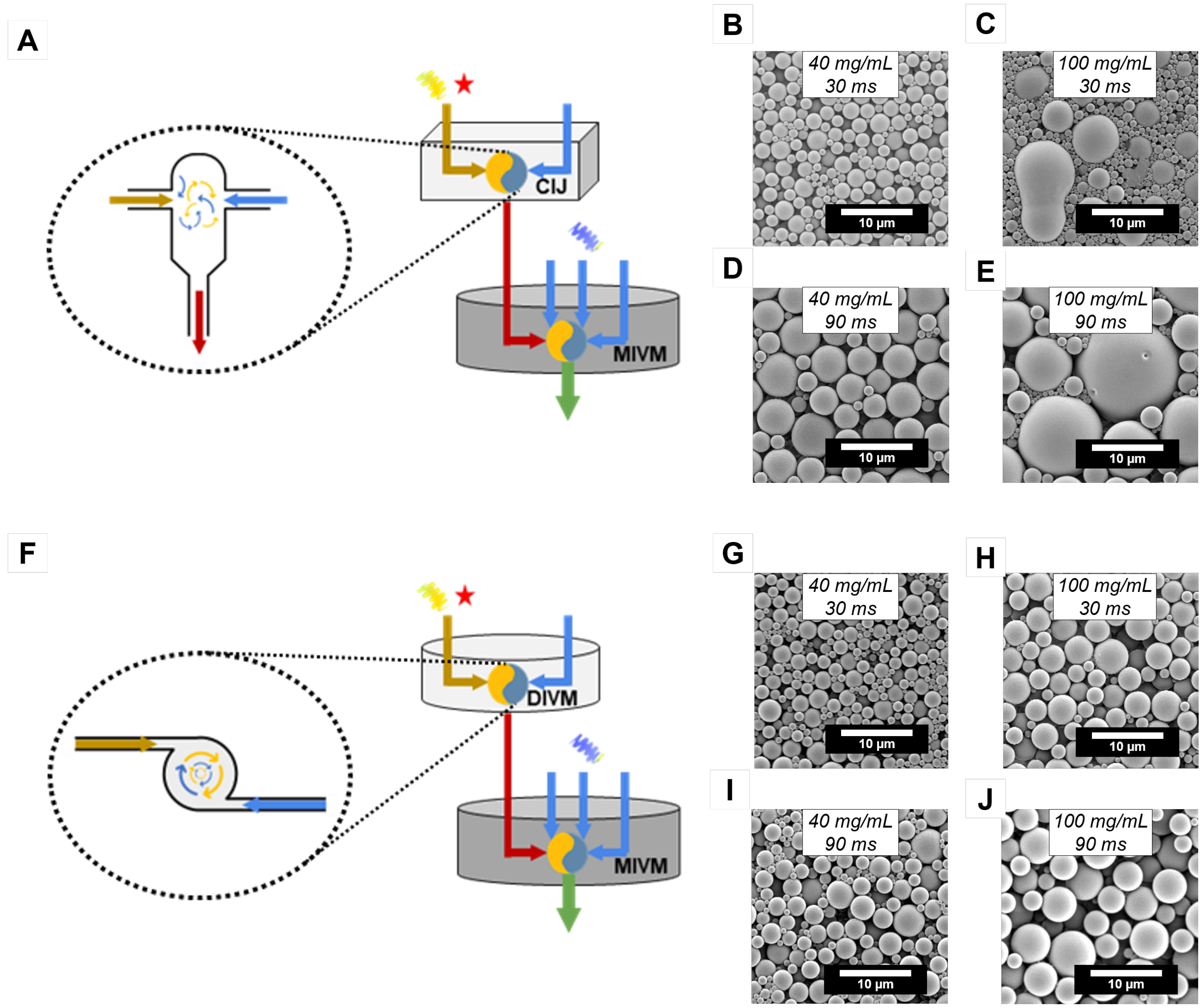

Figure S5. Mixer setup schematics and mixing geometries of **A.** CIJM-MIVM and **F.** DIVM-MIVM mixing geometries. **B-E.** Representative images of MPs synthesized at the labeled C_{core} and T_d for the CIJM-MIVM configuration. **G-J.** Representative images of MPs synthesized at the labeled C_{core} and T_d for the DIVM-MIVM configuration.

In the CIJM-MIVM setup, uniform particle assembly was observed at 40 mg/mL at both short (30ms) and long (90ms) delay times, producing monodisperse 1.9 µm and 3.0 µm particles, respectively (Fig. S5 B&D). However, at 100 mg/mL, severe polydispersity and irregular morphologies emerged, including pear and disc-shaped aggregates—indicators of insufficient mixing during core assembly (Figure S5 C&E).

In contrast, the DIVM-MIVM configuration maintained uniform assembly at both concentrations and both delay times. At 40 mg/mL, 1.6 µm and 2.2 µm particles were synthesized at 30 and 90 ms, respectively (Figure S5 G&I). At 100 mg/mL, 2.9 and 3.0 particles were synthesized at 30 and 90 ms. (Figures S5 H&J).

7. Overlap Concentration of Polylactic Acid Chains in Tetrahydrofuran

Polymer overlap concentration can be calculated from equation S8, where this concentration is the inverse of volume per unit mass of a polymer chain calculated as a spherical globule with radius of gyration R_g.[6] The following calculations resulted in a C* of 111.5 mg/mL.

(8) $\boldsymbol{C^*} \simeq \frac{3M}{4\pi N_A {R_g}^3}$

C* = overlap mass concentration of a polymer in solution

M = molecular weight of single PLA chain, 17.5 kDa

N_A = Avogadro's Number, 6.022 x 10^{23}

This radius of gyration was calculated from equation S9, the mean square radius of gyration of an ideal linear polymer, where N is the number of Kuhn monomers and b is the Kuhn length, calculated from equations S10 and S11, respectively.[2, 7]

(9) $\boldsymbol{R_g^2} = \frac{Nb^2}{6}$

(10) $\boldsymbol{N} = \frac{M}{M_o}$

(11) $\boldsymbol{C_\infty} = \frac{b^2}{n_v l^2}$

C_∞ = Kuhn length characteristic ratio of PLA, 6.5 (Dorgan et al.)[2]

n_v = number of bonds per repeat unit, 6 bonds (Anderson & Hillmyer)[7]

l = length of a repeat unit, 1.41 Å (Anderson & Hillmyer)[7]

M_o = Kuhn monomer weight, 144 Da (Anderson & Hillmyer)[7]

N = # of Kuhn monomers, 122 monomers

B = Kuhn length, 8.81 Å

8. Tabulated Formulation and Size Data

Table S5. Average MP size and PDI from organic stream concentration and delay times tested. (N=3).

***C_{core}* (mg/mL)**	***T_d* (ms)**	***Diameter (µm)***	***PDI***
40	30	1.59 ± 0.01	0.28 ± 0.02
40	60	1.86 ± 0.07	0.12 ± 0.01
40	90	2.24 ± 0.05	0.15 ± 0.05
60	30	1.92 ± 0.09	0.11 ± 0.03
60	60	2.24 ± 0.09	0.17 ± 0.04
60	90	2.57 ± 0.15	0.12 ± 0.05
80	30	2.01 ± 0.20	0.29 ± 0.15
80	60	2.36 ± 0.17	0.27 ± 0.10
80	90	2.87 ± 0.12	0.34 ± 0.09
100	30	2.86 ± 0.11	0.17 ± 0.03
100	60	2.88 ± 0.33	0.24 ± 0.11
100	90	2.98 ± 0.35	0.39 ± 0.15

*Dissolved solids for all formulations contained 2 wt.% rubrene, e.g. core concentration 40 mg/mL corresponds to 39.2 mg/mL PLA and 0.8 mg/mL rubrene. (N = 3). Influence of both Td and Ccore on diameter were calculated to be statistically significant by 2way ANOVA test ($\alpha = 0.5$, $P < 0.0001$ for both parameters).

Table S6. Drug-loaded formulations

***C_{core}* (mg/mL)**	***T_d* (ms)**	***Diameter (µm)***	***PDI***	***EE%***
60	30	1.85 ± 0.25	0.12 ± 0.01	83 ± 6%
60	60	1.69 ± 0.12	0.19 ± 0.05	84 ± 8%
60	90	2.15 ± 0.32	0.31 ± 0.17	85 ± 7%

Dissolved core stream solids for drug-loaded formulations contained 2 wt.% rubrene and 10 wt.% itraconazole, with PLA making up the remaining 88 wt.%. (N = 3)

Table S7. Surface tuning formulations

C_{core} (mg/mL)	T_d (ms)	Diameter (μm)	PDI
40	14	1.06 ± 0.02	0.2 ± 0.07
40	14	1.24 ± 0.01	0.31 ± 0.04
40	14	1.08 ± 0.07	0.19 ± 0.06

*Dissolved solids for all formulations also contained 2 wt.% rubrene. PVA concentration in all three water streams in second mixing step was 1 mg/mL. PVP and PEG-PLA concentrations were 10 mg/mL and 40 mg/mL, respectively. (N = 3)

9. Quantification of Itraconazole via HPLC

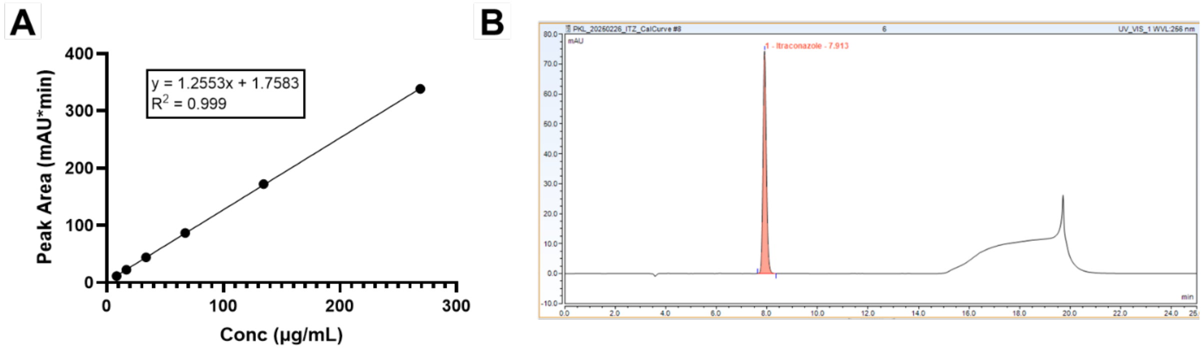

Figure S6. A. High Performance Liquid Chromatography (HPLC) calibration curve for the determination of itraconazole loading into PLA/PVA microparticles via SNAP. **B.** example UV absorbance trace.

10. References

(1) Markwalter, C. E.; Prud'homme, R. K. Design of a Small-Scale Multi-Inlet Vortex Mixer for Scalable Nanoparticle Production and Application to the Encapsulation of Biologics by Inverse Flash NanoPrecipitation. *J Pharm Sci-Us* **2018**, *107* (9), 2465-2471. DOI: 10.1016/j.xphs.2018.05.003.

(2) Dorgan, J. R.; Janzen, J.; Knauss, D. M.; Hait, S. B.; Limoges, B. R.; Hutchinson, M. H. Fundamental solution and single-chain properties of polylactides. *Journal of Polymer Science Part B: Polymer Physics* **2005**, *43* (21). DOI: 10.1002/polb.20577.

(3) Thomas, D. K.; Thomas, T. A. J. Viscosity–concentration relationships in solutions of high polymers. *Journal of Applied Polymer Science* **1960**, *3* (8). DOI: 10.1002/app.1960.070030801.

(4) Baird, Z. S.; Uusi-Kyyny, P.; Pokki, J.-P.; Pedegert, E.; Alopaeus, V. Vapor Pressures, Densities, and PC-SAFT Parameters for 11 Bio-compounds. *International Journal of Thermophysics 2019 40:11* **2019**, *40* (11). DOI: 10.1007/s10765-019-2570-9.

(5) Dukhin, A.; Parlia, S.; Somasundaran, P. Rheology of non-Newtonian liquid mixtures and the role of molecular chain length. *Journal of Colloid and Interface Science* **2020**, *560*. DOI: 10.1016/j.jcis.2019.10.044.

(6) Overlap concentration of macromolecules in solution. *Macromolecules* **1987**, *20* (2). DOI: 10.1021/ma00168a023.

(7) Anderson, K. S.; Hillmyer, M. A. Melt Chain Dimensions of Polylactide. *Macromolecules* **2004**, *37* (5). DOI: 10.1021/ma0357523.
